# Effect of Chronic Stress Present in Fibroblasts Derived from Patients with a Sporadic Form of AD on Mitochondrial Function and Mitochondrial Turnover

**DOI:** 10.3390/antiox10060938

**Published:** 2021-06-10

**Authors:** Karolina Drabik, Dominika Malińska, Karolina Piecyk, Grażyna Dębska-Vielhaber, Stefan Vielhaber, Jerzy Duszyński, Joanna Szczepanowska

**Affiliations:** 1 Nencki Institute of Experimental Biology, Polish Academy of Sciences, Pasteur Street 3, 02-093 Warsaw, Poland; k.drabik@nencki.edu.pl (K.D.); k.piecyk@nencki.edu.pl (K.P.); j.duszynski@nencki.edu.pl (J.D.); 2 Faculty of Chemistry, University of Warsaw, Pasteur Street 1, 02-093 Warsaw, Poland; 3 Department of Neurology, Otto-von-Guericke University of Magdeburg, Universitätsplatz 2, 39106 Magdeburg, Germany; grazyna.debska-vielhaber@med.ovgu.de (G.D.-V.); stefan.vielhaber@med.ovgu.de (S.V.)

**Keywords:** Alzheimer’s disease, mitochondria, reactive oxygen species, mitochondrial biogenesis, mitophagy, mitochondrial retrograde signaling

## Abstract

Although the sporadic form of Alzheimer’s disease (AD) is the prevalent form, the cellular events underlying the disease pathogenesis have not been fully characterized. Accumulating evidence points to mitochondrial dysfunction as one of the events responsible for AD progression. We investigated mitochondrial function in fibroblasts collected from patients diagnosed with the sporadic form of AD (sAD), placing a particular focus on mitochondrial turnover. We measured mitochondrial biogenesis and autophagic clearance, and evaluated the presence of bioenergetic stress in sAD cells. The mitochondrial turnover was clearly lower in the fibroblasts from sAD patients than in the fibroblasts from the control subjects, and the levels of many proteins regulating mitochondrial biogenesis, autophagy and mitophagy were decreased in patient cells. Additionally, the sAD fibroblasts had slightly higher mitochondrial superoxide levels and impaired antioxidant defense. Mitochondrial turnover undergoes feedback regulation through mitochondrial retrograde signaling, which is responsible for the maintenance of optimal mitochondrial functioning, and mitochondria-derived ROS participate as signaling molecules in this process. Our results showed that in sAD patients cells, there is a shift in the balance of mitochondrial function, possibly in response to the presence of cellular stress related to disease development.

## 1. Introduction

The characteristic hallmarks of Alzheimer’s Disease (AD) are amyloid β (Aβ) accumulation, and tau protein hyperphosphorylation, which lead to the formation of amyloid plaques and neurofibrillary tangles in the brain tissue. These pathologies have long been considered the main mechanisms underlying disease development [1,2,3]. However, the failure of therapies directed against Aβ accumulation [4,5,6], and more intensive investigation of cellular events underlying AD development, led to the formulation of alternative hypotheses, considering mitochondrial dysfunction [7,8], the dysregulation of calcium homeostasis [9], and auto and mitophagy impairment [10] as the triggers of disease development. It was also proposed that some of those disturbances can form vicious cycles, where different cellular dysfunctions reciprocally aggravate each other [11,12]. Therefore, further intensive research needs to be conducted to better explain the cellular events leading to AD development and the causal relationships between them.

The sporadic form (sAD) is the most common form of AD. The causative genetic defects for several familial forms of AD (fAD) have been identified; however, the etiology of sporadic AD remains unknown. Although the pathogenesis of AD is not well understood, accumulating evidence implicates mitochondrial dysfunction as an important event in the disease progression.

Mitochondria are organelles with a multitude of functions, ranging from energy production to participation in intracellular signaling, which regulates cellular metabolism, energy homeostasis, stress response, and cell fate [13]. Under conditions of altered environmental demands, the reprogrammed signaling in mitochondria plays a crucial role in maintaining metabolic flexibility [12,14]. Mitochondria adapt to a wide range of cellular requirements, adjusting fuel utilization, meeting ATP requirements, regulating reactive oxygen species (ROS) output, and titrating the redox balance of the cell. The changes in mitochondrial function are reflected in intracellular levels of ATP, ROS or Ca^2+^, which act as carriers of information in mitochondria-to-nucleus communication, known as mitochondrial retrograde signaling [15]. In response, the expression of the nuclear genes responsible for the feedback regulation of mitochondrial function is altered. This triggers cytosolic changes regulating the mitochondrial network morphology, mitochondrial biogenesis, mitophagy and other events adjusting mitochondrial function, hereby supporting cellular adaptation or cell death [16,17]. Mitochondrial biogenesis and removal via mitophagy define the mitochondrial turnover rate and are crucial for the maintenance of a properly functioning mitochondrial pool. Thus, these processes are strongly affected in response to mitochondrial stress and mitochondrial retrograde signals. 

Because mitochondrial stress remodeling is a component of the pathophysiology of diverse neurological diseases, and our knowledge of the pathogenesis and pathophysiology of sAD is still insufficient, we investigated the effect of mitochondrial signaling and adaptation to chronic stress in sAD. Our experimental model was fibroblasts collected from patients diagnosed with sAD. We examined the levels of the following mediators involved in the response to chronic stress: indicators of the mitochondrial biogenesis process, autophagy and mitophagy. We also checked the intensity of mitochondrial turnover, as changes in mitochondrial turnover are intimately linked to the maintenance of cell homeostasis via the adaptation of vital functions. We observed slightly higher levels of mitochondrial superoxide and simultaneously modified levels of enzymes of the antioxidative system. In response to stress, there was also a change in the regulation of mitochondrial biogenesis, autophagic removal and turnover, with a greater population of old mitochondria present in sAD fibroblasts. Our measurements revealed alterations in mitochondrial function accompanying sAD-related cellular stress.

The lack of effective means to prevent or treat AD, and the failure of recent clinical trials, emphasize the need for a better understanding of sAD pathogenic mechanisms to find novel targets for AD therapeutic interventions.

## 2. Materials and Methods

### 2.1. Chemicals and Antibodies

Ionomycin, antimycin A, carbonyl cyanide 3-chlorophenylhydrazone (CCCP), H_2_O_2_, protease and phosphatase inhibitors, the antibiotics penicillin and streptomycin, ciprofloxacin, tylosin and Tween 20 were purchased from Sigma-Aldrich (St. Louis, MO, USA). Odyssey blocking buffer was obtained from Li-Cor Bioscience (Li-Cor Bioscience, Lincoln, NE, USA). DMEM was obtained from PAN-Biotech GmbH (Aidenbach, Germany), and FBS was purchased from Gibco (Grand Island, NY, USA). The MitoTimer vector was obtained from Addgene, and the transfection reagent TransfeX was purchased from ATCC (Manassas, VA, USA). Live imaging medium (LIM), fluorescent probes fluo-4-AM, CM-H_2_DCFDA, MitoSox Red, MitoTracker Green FM and JC-1 were purchased from Thermo Fisher Scientific (Waltham, MA, USA). All other chemicals were of analytical grade.

Primary antibodies against polymerase γ (POLG), catalase, parkin and rabbit anti-β-actin were obtained from Abcam (Cambridge, UK); antibodies against mitochondrial transcription factor A (TFAM), superoxide dismutase 2 (SOD2), Beclin-1, LC3 and p62 were obtained from Cell Signaling Technology (Danvers, MA, USA); antibodies against nuclear respiratory factors 1 and 2 (NRF1 and NRF2) were purchased from Proteintech (Manchester, UK); antibodies against PTEN-induced kinase 1 (PINK1) were procured from Novus Biologicals (Centennial, CO, USA); antibodies against superoxide dismutase 1 (SOD1) were purchased from Santa Cruz Biotechnology (Dallas, TX, USA); mouse anti-β-actin was obtained from Sigma-Aldrich (St. Louis, MO, USA). The IRDye680 and IRDye800 secondary antibodies were purchased from Li-Cor Biosciences (Bad Homburg, Germany). For Western blotting, secondary antibodies were diluted 1:10,000 and primary antibodies were used at a dilution of 1:1000, except for anti-SOD1 and anti-SOD2 (1:2000), mouse anti-β-actin (1:400,000), and rabbi anti-β-actin (1:400,000).

### 2.2. Patients and Control Individuals

The research was conducted using human primary fibroblasts derived from skin biopsies from four patients diagnosed with sAD and four control donors (Table 1). The fibroblasts from the sAD patients and those from the control donor C4 were derived from the Department of Neurology, Otto von Guericke’s University of Magdeburg (Germany), while controls C1–C3 were obtained from the cell repository of the Coriell Institute for Medical Research (repository numbers ND34769, ND36320 and ND36091, respectively). All patients were diagnosed at the Department of Neurology of University of Magdeburg, based on their disease phenotype and scores according to the Folstein Mini-Mental State (MMS) protocol. The maximal MMS score is 30, and the average score given to healthy individuals aged older than 60 years is approximately 26–27, while the MMS score of the sAD fibroblast donors included in the study ranged between 20 and 23, denoting mild cognitive and affective impairments. This study was approved by the ethical committee of the University of Magdeburg, and written informed consent was obtained from all of the subjects.

### 2.3. Primary Fibroblast Culture

Fibroblasts were cultured at 37 °C in a humidified atmosphere containing 5% carbon dioxide in high-glucose DMEM supplemented with 10% heat-inactivated fetal bovine serum (FBS) and the following antibiotics: 100 U/mL penicillin, 100 μg/mL streptomycin, 4 μg/mL ciprofloxacin and 10 μg/mL tylosin. All experiments were repeated at least three times using cells between the 3rd and 10th passages.

### 2.4. Live Cell Studies 

Cells were seeded onto 24-well plates at a density of 15,000 cells per well and grown for 24 h. On the day of the experiment, the cells were loaded with the appropriate fluorescent probes, as follows: 2 µM fluo-4-AM for determination of cytosolic Ca^2+^ levels, 5 µM H_2_DCF-DA for cytosolic ROS measurements, 5 µM MitoSOX Red for mitochondrial superoxide detection, 200 nM MitoTracker Green FM for mitochondrial mass determination, or 1.5 µM JC-1 for measurements of mitochondrial membrane potential. Loadings with JC-1 and MitoTracker Green were conducted in culture media, and all other probes were loaded in PBS containing 0.9 mM CaCl_2_ and 0.49 mM MgCl_2_. After 30 min of incubation at 37 °C, the probe was removed by 3 washes with PBS, fresh PBS with Ca^2+^ and Mg^2+^ was added to the wells, and the cells were visualized with an iCys laser scanning cytometer (LSC) (Thorlabs Inc., Newton, NJ, USA) using a 20× objective. Probes with green fluorescence (fluo-4-AM, H_2_DCF-DA, MitoTracker Green FM and JC-1) were excited at 488 nm, and the emission was collected using a 530/30 nm filter cube. Red fluorescence of JC-1 and MitoSox Red were collected through a 580/30 nm filter cube, upon excitation with a 488 nm laser. The measurements were verified with the appropriate positive controls included in the experiments, as follows: treatment with 1 µM ionomycin for cytosolic Ca^2+^ determination, 1 mM H_2_O_2_ for cytosolic ROS measurements, 2 µg/mL antimycin A for mitochondrial superoxide detection and 5 µM CCCP for mitochondrial membrane potential determination. Data analysis was performed with iCys software version 2.5 using the phantom contours approach, as follows: each microscopy image was divided into a grid of smaller regions (phantom contours) in which the fluorescent signal was quantified. Then, the fluorescence threshold was set, which separated the phantoms representing the cells and the background, and based on that, the integrated fluorescence signal coming from the analyzed cells was quantified for each well. In each experiment, three technical replicates were performed for each of the cell lines. The results were normalized to the average value obtained in the particular experiment for controls C1 and C2, which were the reference controls, included in each of the analyzed 24-well plates.

### 2.5. Whole-Cell Extracts and Immunoblotting

Cells growing on 10 cm diameter dishes or in 75 cm^2^ culture flasks were harvested with 0.05% trypsin, washed with ice-cold PBS, sedimented by centrifugation and resuspended in RIPA lysis buffer supplemented with protease (1:100) and phosphatase inhibitor (1:100) cocktails. After 30 min of incubation on ice, the lysates were centrifuged at 16,000× *g* and 4 °C for 20 min. The supernatants were collected, and the protein concentration was determined according to the Bradford method. The samples were then mixed with loading buffer (0.5 M Tris-HCl, pH 6.8, 2.3% SDS, 5% mercaptoethanol (*v/v*), 12.5% glycerol (*v/v*) and 0.04% bromophenol blue) and heated for 5 min at 95 °C. The prepared lysates were resolved by SDS-PAGE, transferred onto nitrocellulose membranes and blocked for 1 h with Odyssey^®^ blocking buffer diluted 1:1 in TBS (20 mM Tris, pH 7.6 and 150 mM sodium chloride). Subsequently, the membranes were incubated in 1:1 Odyssey blocking buffer:TBS buffer supplemented with Tween 20 (0.1%) with appropriate primary antibodies overnight at 4 °C. After washing with TBS 0.1% Tween buffer (TBS-T), the membranes were incubated with secondary antibodies (IRDye antibodies) for 1 h at room temperature. The blots were rinsed with TBS-T and visualized using the Odyssey infrared imaging system (Li-Cor Biosciences, Lincoln, NE, USA). Image Studio Lite was used for the densitometric analysis. After correction for the loading control (β-actin band intensity), the results were normalized to the average value obtained in the particular experiment for controls C1 and C2, which were reference controls, included in each of the analyzed Western blot membranes.

### 2.6. Visualization of Mitochondrial Age with MitoTimer Vector

Cells were seeded in 24-well plates at a density of 10,000 cells per well and grown under standard culture conditions. After 24 h, the cells were transfected with the MitoTimer vector using the TransfeX kit in accordance with the manufacturer’s recommendations. Twenty-four hours after transfection, the cells were imaged by confocal microscopy (Zeiss spinning disc microscope) using an HC APO 63×/1.20 water objective. To ensure a constant molecular brightness of the MitoTimer, the following same settings (resolution: 512 × 512 format with a pixel size of 132 nm) were used:

- Green fluorescence: excitation/emission—490/500–540 nm, EM gain—600, exposure time—750 ms, laser power—4%;
- Red fluorescence: excitation/emission—550/580–640 nm, EM gain—700, exposure time—150 ms, laser power—4%.

Pictures were taken from randomly selected fields with a Z-stack section of 0.6 µm.

Quantification of the MitoTimer fluorescence was performed with ImageJ software. First, a mask was created covering all regions presenting green and/or red fluorescence, corresponding to the mitochondrial network. Furthermore, the mask was applied to extract mitochondria from the original image, the red and green fluorescence was quantified within the masked region, and the green-to-red fluorescence ratio was calculated. For each cell line at least 40 cells were analyzed. 

### 2.7. Statistical Analysis

In the experiments using Western blot or laser scanning cytometry analysis, statistical significance was assessed using Student’s *t*-test to compare the results obtained for the control (*n* = 4) and sAD (*n* = 4) fibroblast cell lines. In the microscopic study, where single-cell analysis was performed, Student’s *t*-test was applied to compare the results obtained for the individual fibroblast cell lines. Only *p* values lower than 0.05 were considered to be statistically significant. 

## 3. Results

### 3.1. Fibroblasts from sAD Patients Show Signs of Increased Cellular Stress

**Figure 1:**
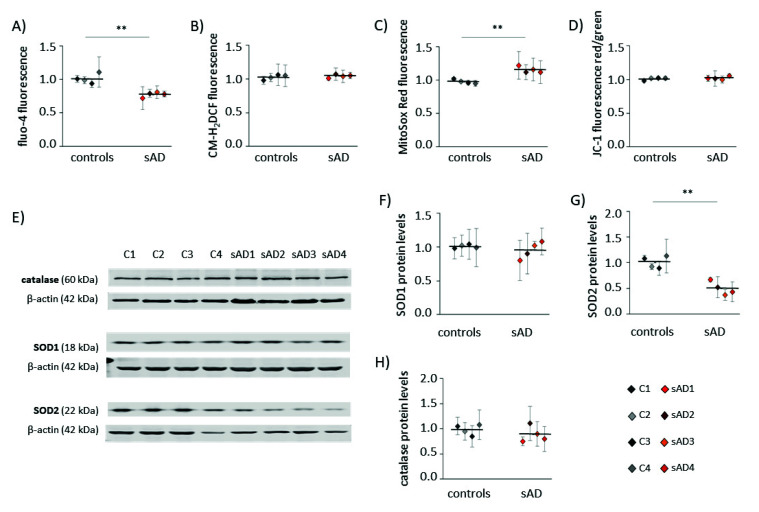
Hallmarks of cellular stress in primary fibroblasts from sAD patients. (**A**) Cytosolic Ca^2+^ levels measured with fluo-4-AM. (**B**) Cytosolic ROS levels measured with CM-H_2_DCFDA. (**C**) Superoxide levels in mitochondria determined with MitoSOX Red. (**D**) Mitochondrial membrane potential measured with JC-1. (**E**) Representative results of Western blot analysis of the antioxidant enzymes levels in the investigated fibroblasts. The results of quantification based on at least three independent repetitions (three independent cell lysates for each cell line) are presented in the following panels: (**F**) for SOD1, (**G**) for SOD2 and (**H**) for catalase. All of the results were normalized to the average levels measured for the reference controls C1 and C2 in the particular experiment. The results for individual cell lines are presented as the means ± SDs from 3–7 independent experimental repetitions. Thick black lines show averaged values for the analyzed groups: the controls and the sAD fibroblasts. Asterisks denote statistical significance: * *p* < 0.05, ** *p* < 0.01 in Student’s *t*-test comparing controls (*n* = 4) vs. sAD (*n* = 4).

An increasing number of reports have shown that mitochondrial dysfunction is an early event in AD development [10,18,19] and that this dysfunction is not limited to neurons, but may also be observed in the peripheral tissues of AD patients [20,21,22,23,24]. Alterations in mitochondrial homeostasis affect multiple aspects of cellular function, including calcium handling, redox homeostasis and metabolism. In the investigated control and sAD fibroblasts, we analyzed the cytosolic levels of Ca^2+^ and ROS (Figure 1A,B). Both of these parameters are reciprocally related to mitochondrial function, as they are affected by changes in mitochondrial activity, and are, at the same time, carriers of information on the mitochondrial functional state in mitochondria-to-nucleus communication (mitochondrial retrograde signaling) [25]. In the investigated sAD fibroblast lines, the cytosolic Ca^2+^ was 23 ± 3.8% lower than that in the controls, while the cytosolic ROS appeared unaltered. We also analyzed the following parameters that are directly related to mitochondrial function: mitochondrial membrane potential (ΔΨ) and mitochondrial superoxide levels (Figure 1C,D), which both tightly reflect the mitochondrial functional state. The ΔΨ was comparable in all of the investigated cell lines, but we detected a slight increase in mitochondrial superoxide in the sAD fibroblasts (on average 18 ± 4.8% higher than in the controls). It was accompanied by clearly lower levels of SOD2 (50 ± 13.0% decrease), which is responsible for the superoxide removal from the mitochondrial matrix. There were no changes in the levels of catalase or SOD1, which is present in the cytosol and in the mitochondrial intermembrane space (Figure 1E–H).

### 3.2. Mitochondrial Turnover in sAD Fibroblasts Is Slower Than That in Control Cells

**Figure 2:**
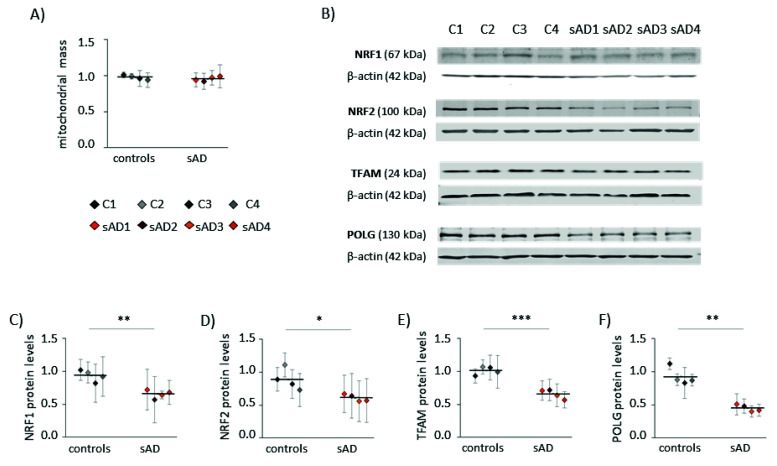
Markers of mitochondrial biogenesis in the investigated fibroblasts. (**A**) Mitochondrial mass determined with MitoTracker Green fluorescent probe. (**B**) Representative result of Western blot analysis of the mitochondrial biogenesis markers levels in the investigated fibroblasts. The results of quantification based on at least three independent repetitions (three independent cell lysates for each cell line) are presented in the following panels: (**C**) for NRF1, (**D**) for NRF2, (**E**) for TFAM and (**F**) for POLG. All of the results were normalized to the average levels measured for the reference controls C1 and C2 in each particular experiment. The results for individual cell lines are presented as the means ± SDs from 3–6 independent experimental repetitions. Thick black lines show the averaged values for the analyzed groups: the controls and the sAD fibroblasts. Asterisks denote statistical significance: * *p* < 0.05, ** *p* < 0.01, *** *p* < 0.001 in Student’s *t*-test comparing controls (*n* = 4) vs. sAD (*n* = 4).

Increased levels of mitochondrial superoxide may result not only from changes in mitochondrial function, but also from elevated mitochondrial content in the cell. Increased mitochondrial biogenesis is often a response to bioenergetic stress, aiming to compensate for the decreased efficiency of ATP production [26]. However, in the analyzed fibroblasts, the mitochondrial mass did not differ between sAD and the controls (Figure 2A). Moreover, in the sAD cells, we observed lower levels of the following proteins involved in mitochondrial biogenesis: mitochondrial DNA polymerase POLG and mitochondrial transcription factor TFAM, activating the transcription of mitochondria-encoded genes (lower than in the controls by 51 ± 5.5% and 35 ± 6.9%, respectively—Figure 2E,F). Additionally, nuclear transcription factors NRF1 and NRF2, coordinating mitochondrial biogenesis, were slightly decreased in the sAD cells compared to the controls (by 30 ± 6.8% and 31 ± 6.0%, respectively—Figure 2C,D).

**Figure 3:**
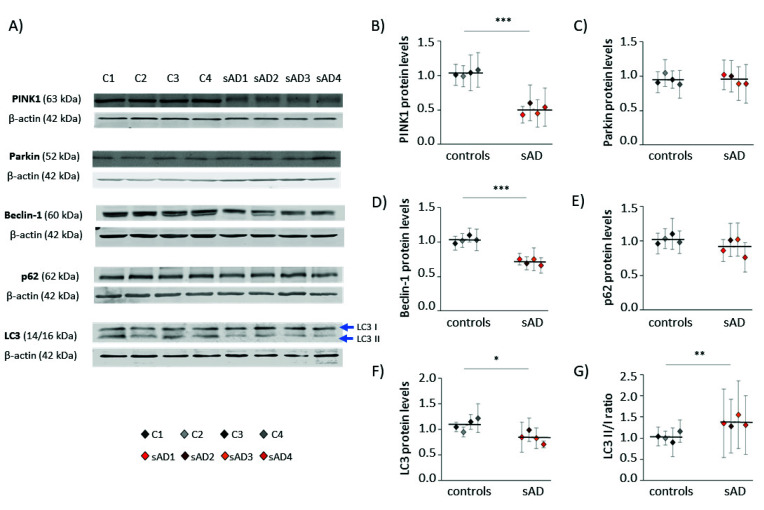
Levels of mitophagy effectors in the investigated fibroblasts. (**A**) Representative results of Western blot analysis of the levels of proteins involved in autophagy and mitophagy in the investigated fibroblasts. The results of quantification performed in at least three independent repetitions (with independent cell lysates) are presented in the following panels: (**B**) for PINK1, (**C**) for Parkin, (**D**) for Beclin-1, (**E**) for p62, and (**F**) for LC3. (**G**) The ratio of LC3 II/I forms. All of the results were normalized to the average levels measured for the reference controls C1 and C2 in each particular experiment. The results for individual cell lines are presented as the means ± SDs from 3–9 independent experimental repetitions. Thick black lines show averaged values for the analyzed groups: the controls and the sAD fibroblasts. Asterisks denote statistical significance: * *p* < 0.05, ** *p* < 0.01, *** *p* < 0.001 in Student’s *t*-test comparing controls (*n* = 4) vs. sAD (*n* = 4).

The amount of mitochondria in the cell is the result of opposing processes of mitochondrial biogenesis and mitophagy [25]. Thus, we also checked the levels of proteins involved in the process of autophagy, as well as those specific for mitophagy—the selective autophagy of mitochondria (Figure 3). Many of them turned out to be decreased in sAD fibroblasts when compared to the control cell lines; the levels of PINK1 were lowered by 51 ± 7.7%, of Beclin-1 by 31 ± 4.3%, and of LC3 by 23 ± 11%. These proteins act at different stages of mitochondrial removal by mitophagy, as follows: PINK1 mediates the labeling of dysfunctional mitochondria directed for removal, while Beclin-1 and LC3 coordinate autophagosome formation [26,27]. 

**Figure 4:**
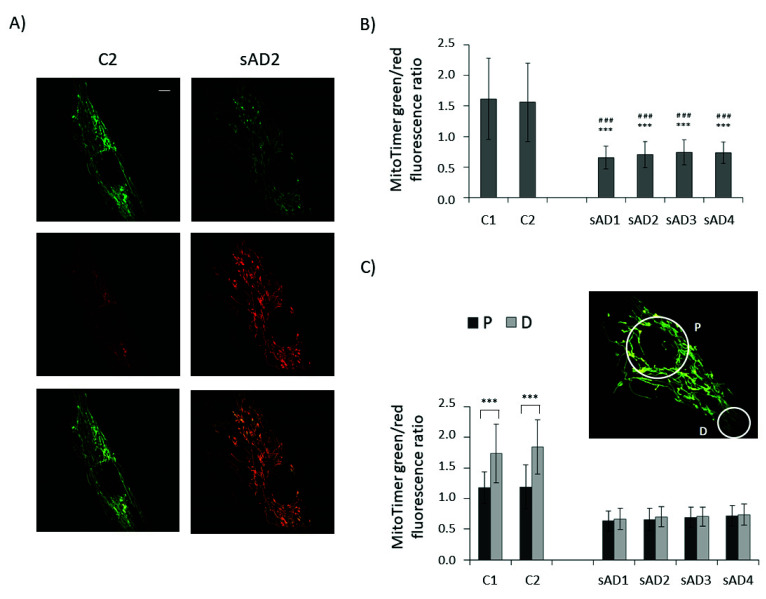
Mitochondrial age in the investigated fibroblasts. (**A**) Representative images of green and red MitoTimer fluorescence and overlaid images for control and sAD fibroblasts, 24 h after MitoTimer transfection. Scale bar refers to 5 µm. (**B**) MitoTimer green/red fluorescence ratio, indicative of mitochondrial age in the analyzed fibroblasts. The graphs present means ± SD from the analysis of *n* = 40–53 cells for each cell line, captured in three independent experiments (independent transfections). Asterisks denote statistical significance in Student *t*-test: *** *p* < 0.001 vs. C1, ### *p* < 0.001 vs. C2. (**C**) The comparison of the MitoTimer green/red fluorescence ratios in perinuclear areas (P) and in distal parts of the cells (D) in the investigated cell lines. Asterisks denote statistical significance: *** *p* < 0.001 in Student’s *t*-test comparing MitoTimer fluorescence ratio in perinuclear and distal regions of the cell in the particular fibroblast cell line. The inset presents the localization of the cellular regions, between which MitoTimer fluorescence ratios were compared.

To check whether lower levels of proteins involved in both mitochondrial biogenesis and mitophagy translate to slower turnover of the mitochondrial pool, we checked the age of the mitochondria in the investigated fibroblasts with the use of the MitoTimer vector (Figure 4). It allows us to assess the age of individual mitochondria based on the extent of the fluorescence shift from green to red, accompanying the maturation of mitochondria-targeted fluorescence protein [28,29,30]. In line with the observed decreases in the levels of mitochondrial biogenesis and removal effectors, the turnover of mitochondria was slower in the sAD fibroblasts, as the age of the mitochondria in the sAD cells was clearly higher than in the controls. The mitochondrial life cycle is also associated with the distribution of active mitochondria in different regions of the cell and with transport of dysfunctional organelles toward perinuclear areas, where they undergo mitophagy [31]. Therefore, we compared the mitochondrial age in perinuclear regions with that in distal parts of the cells. In the control fibroblasts, the mitochondria in the cell peripheries were significantly younger than in the region surrounding the cell nucleus. In the sAD fibroblasts, such a gradient of mitochondrial age was not observed, showing that the regulation of mitochondrial removal in sAD patients’ cells is altered not only by decreased levels of the responsible proteins, but also at the level of mitochondrial transport toward the places of their removal.

## 4. Discussion

The sporadic form of Alzheimer’s disease is much more prevalent, but also far less studied than the familial form (fAD). Despite similar symptoms, the cellular events underlying the development of the sporadic and familial forms of neurodegenerative diseases may considerably differ [32,33,34,35]. The problem in investigating sAD is the limited availability of study models, which is caused by the poorly described and multifactorial etiology of the disease, in contrast to fAD, which is caused by known genetic mutations, for which several animal models are described [32]. A valid animal model of sAD is lacking. Additionally, therapies with promising results in preclinical tests in fAD animal models regularly fail in clinical trials, underlining the necessity to further investigate the differences between fAD and sAD [32,36]. 

Currently, sAD-related mechanisms are studied in amyloid-treated cell cultures, postmortem brain specimens, AD patient fibroblasts, and fibroblast-derived induced pluripotent stem (iPS) cells, which further differentiate to neurons [19,32,33]. In our study, we used primary dermal fibroblasts from sAD patients. It was demonstrated that AD-related pathological changes are not limited to neurons, but can also be observed in peripheral cells, including fibroblasts [23,37]. In contrast to postmortem brain specimens, fibroblast cultures allow us to investigate the earlier stages of disease development and to analyze vital cellular processes. They also complement iPS-based research, as iPS cells may lose some of the AD-characteristic features and markers due to complex reprogramming processes.

Our results point to the presence of cellular stress in sAD fibroblasts, demonstrated as alterations in Ca^2+^ homeostasis, increased mitochondrial ROS production and decreased mitochondrial turnover, resulting in more aged mitochondria. The existence of chronic stress in sAD fibroblasts has already been proposed upon analysis of the gene expression profile [38]. 

We detected lower cytosolic Ca^2+^ levels in the sAD fibroblasts than in the controls, revealing dysregulations within intracellular Ca^2+^ homeostasis. The literature data concerning intracellular Ca^2+^ levels in AD are not consistent. In fAD models, elevated cytosolic Ca^2+^ is often observed; however, a decrease was also reported, depending on the type of fAD-causing mutation [9]. Data concerning intracellular Ca^2+^ handling in sAD are scarce; however, a decrease in cytosolic Ca^2+^ [39] and blunted calcium fluxes in response to various treatments [40] have already been reported for sAD fibroblasts. The discrepancies between fAD and sAD models concerning calcium homeostasis underline the need for valid experimental setups for sAD investigation, as extrapolation of the observations from fAD models to sAD cannot always be done.

The sAD fibroblasts presented with elevated levels of mitochondrial superoxide. Despite that, we did not observe an increase in cytosolic ROS, in contrast to what was previously reported for fAD [41] and sAD fibroblasts [42]. On the other hand, the experiments on iPSC-derived neurons from sAD patients demonstrated strong heterogeneity with respect to cytosolic ROS levels in cell lines from different donors. In addition to sAD cell lines, with clearly increased cytosolic ROS, there were also the lines with ROS levels not differing from the controls [43]. 

The increase in mitochondrial superoxide levels was accompanied by lower levels of mitochondrial superoxide dismutase (SOD2), responsible for superoxide removal from the mitochondrial matrix. SOD2 protein levels are regulated by several mechanisms, at the transcriptional stage as well as post-transcriptionally [44,45]. In neurodegenerative diseases, an increase rather than a decrease in SOD2 is often observed, which has been interpreted as a compensatory mechanism aiming to counterbalance the increased mitochondrial superoxide production [46]. However, in our model, the increase in mitochondrial superoxide was moderate and did not cause extensive mitochondrial damage. The mitochondrial membrane potential remained unaltered, indicating well-maintained mitochondrial function. 

Mitochondrial ROS play a dual role in the cell. On the one hand, their excess can be deleterious, leading to oxidative damage to mitochondrial proteins and DNA, and to oxidative stress. On the other hand, when produced at moderate levels, mitochondrial ROS participate in intracellular signaling, for example, in mitochondria-to-nucleus communication [47,48]. Such retrograde signaling passes information on the mitochondrial functional state to the nucleus and triggers adaptive responses, adjusting mitochondrial function in response to changing extracellular and intracellular conditions [48]. One of the responses regulated by mitochondrial retrograde signaling is mitochondrial dynamics and turnover [16].

Mitochondria form a dynamic network shaped by fusion and fission events, mitochondrial transport along the cytoskeleton, and mitochondrial turnover mediated by mitochondrial biogenesis and mitophagy [49]. The role of these processes is the maintenance of a properly functioning mitochondrial pool and the adjustment of mitochondrial function to changing conditions. 

In sAD fibroblasts, mitochondrial turnover turned out to be much slower than in the controls; the mitochondrial mass remained unaltered, but the age of the mitochondria was clearly higher in the sAD cells. Our finding is in line with the work of Martin-Maestro et al., which demonstrated a slower exchange of the mitochondrial pool in sAD fibroblasts after CCCP treatment [37]. The velocity of mitochondrial pool renewal is the result of mitochondrial biogenesis and mitochondrial clearance by mitophagy [25]. The levels of proteins involved in both biogenesis and mitophagy were lower in the sAD fibroblasts than in the controls, indicative of a lower activity of those processes. Similar findings were reported for postmortem hippocampal samples from AD patients, in which decreased levels of both mitochondrial biogenesis regulators [50,51] and some of the proteins involved in mitophagy [52] were observed. 

The main regulator of mitochondrial biogenesis is the transcription coactivator PGC-1α, inducing the expression of the transcription factors NRF1 and NRF2, which in turn stimulate the expression of nuclear-encoded mitochondrial proteins, such as respiratory chain proteins and the mitochondrial transcription factor TFAM, responsible for mtDNA gene expression, and POLG, mediating mtDNA replication [53]. NRF2 is also involved in the oxidative stress response [54]. We observed slightly decreased levels of NRF1 and NRF2, and a clear downregulation of TFAM and POLG, which indicates slower mitochondrial biogenesis in the sAD fibroblasts than in the controls.

Mitophagy is selective autophagy, directed toward the removal of dysfunctional mitochondria. The best described mitophagy mechanism is the PINK1–Parkin-dependent pathway, directing defective mitochondria to autophagy based on their decreased membrane potential [10]. In sAD fibroblasts, we detected decreased levels of proteins participating in autophagy (Beclin-1, p62 and LC3), as well as of PINK1, which is involved in the selective directing of mitochondria to autophagic removal. There is growing evidence that mitophagy impairment plays an important role in the pathogenesis of AD, as well as of some other neurodegenerative diseases [10]. Bidirectional dependencies have been demonstrated between the efficiency of mitophagy and the accumulation of the Aβ or tau hyperphosphorylation, which may lead to the formation of vicious cycles accelerating disease progression [10]. Thus, mitophagy is considered a promising therapeutic target for AD. In mouse models of fAD, the stimulation of mitophagy not only decreased the number of damaged mitochondria in the brain, but also attenuated Aβ deposition and tau hyperphosphorylation, and ameliorated cognitive impairment [52]. 

Mitochondrial turnover strongly depends on other components of mitochondrial dynamics, such as mitochondrial transport, fusion and fission. Mitochondrial fission allows for the separation of defective parts of the mitochondrial network, which are then directed for autophagic removal. In turn, mitochondrial network hyperfusion can protect mitochondria from excessive mitophagy, for example, during starvation stress [49]. Mitochondrial transport plays an important role in regulating mitochondrial turnover, as the main sites of mitochondrial clearance are located in perinuclear areas [27]. Thus, before being removed, damaged mitochondria need to be transported from the cell periphery toward the perinuclear regions. In the control fibroblasts, there was a clear difference in the age of the mitochondria in the perinuclear area and in the distant parts of the cell, which is in line with aged mitochondria being transported toward the cell nucleus. In sAD fibroblasts, the age of the mitochondria did not differ between the perinuclear and distal parts of the cell, which shows that not only the mitophagic clearance of old mitochondria, but also the mechanisms of mitochondrial transport within the cell are defective. Indeed, in our previous work we detected decreased mitochondrial transport velocity, a lower frequency of fusion–fission events and a more fused mitochondrial network in sAD fibroblasts [55]. All of these events can contribute to impairments in mitochondrial turnover. 

Decreased efficiency of mitochondrial fusion, fission, transport and turnover presents an image of generally slower mitochondrial dynamics in the fibroblasts of sAD patients. This may be a hallmark of a decrease in the cellular metabolic rate accompanying the disease process. Despite that, we did not observe any symptoms of increased cell death in the sAD fibroblasts, their cell morphology was maintained, and only their slower proliferation rates could be a hallmark of the decreased metabolic activity of sAD cells [55]. However, it should be noted that fibroblasts adapt to bioenergetic deficits much more readily than neurons, for which such a degree of mitochondrial disturbances can lead to more severe cellular dysfunction. The literature data confirm that mitochondrial dynamics are also impaired in sAD brains; for example, in postmortem brain samples of sAD patients, decreased expression of genes related to mitochondrial turnover, fusion and fission was demonstrated [37]. This confirms that many pathological processes occurring in sAD patient brains are also reflected in fibroblasts.

## 5. Conclusions

The data collected with our model show an overall decrease in mitochondrial dynamics in sAD fibroblasts. Both mitochondrial biogenesis and mitophagy were reduced in the sAD cells, resulting in slower mitochondrial turnover and increased mitochondrial age. Additionally, the mitochondrial superoxide levels in the sAD cells were increased, accompanied by lower levels of SOD2. What remains unclear is what the causal relationships between the different aspects of cellular homeostasis disruption observed in the sAD fibroblasts are, and what the primary trigger of those changes is. Increased mitochondrial age could be the cause of the elevated mitochondrial superoxide production, as older mitochondria tend to produce more ROS [56,57]. Higher mitochondrial ROS are a hallmark of less efficient mitochondrial functioning, which, however, did not lead to extensive mitochondrial damage; the mitochondrial membrane potential remained unaltered, reflecting efficient compensation for the increased mitochondrial age in the fibroblasts. Slower mitochondrial biogenesis is counterbalanced by a decreased mitophagy efficiency, which allows for the maintenance of an unchanged mitochondrial mass, even at the cost of a higher age of the mitochondria and a slight impairment of their function. It is possible that chronic cellular stress, present in fibroblasts, leads to the remodeling of mitochondrial retrograde signaling to keep the cell alive in the best condition possible. Such an adaptive scenario is sufficient in the case of fibroblasts, where feedback adjustment of mitochondrial function to the occurring bioenergetic deficiencies leads to setting a new balance, enabling cell survival despite stress conditions. In postmitotic tissues, with a higher dependence on oxidative phosphorylation, such as neural tissue, such alterations in mitochondrial function may be deleterious. Currently, the evidence is accumulating supporting the hypothesis that impaired mitochondrial turnover, particularly defective mitophagy, plays a crucial role in the pathogenesis of several neurodegenerative diseases, including AD [58]. Mitophagy is thus one of the focus points in searching for efficient AD treatments [10,53].

## Figures and Tables

**Table 1:** Primary fibroblasts used in the study—donors data.

Symbol	Sex ^1^	Age
**sAD patients**		
sAD1	M	74
sAD2	F	74
sAD3	M	83
sAD4	F	71
**Controls**		
C1	F	68
C2	F	71
C3	F	63
C4	F	67

^1^ F—female, M—male.

## Data Availability

All relevant data are presented in this manuscript.

## References

[1] Area-Gomez E., Schon E.A. (2017). Alzheimer Disease. Adv. Exp. Med. Biol..

[2] Dos Santos Picanco L.C., Ozela P.F., de Fatima de Brito Brito M., Pinheiro A.A., Padilha E.C., Braga F.S., de Paula da Silva C.H.T., Dos Santos C.B.R., Rosa J.M.C., da Silva Hage-Melim L.I. (2018). Alzheimer’s Disease: A Review from the Pathophysiology to Diagnosis, New Perspectives for Pharmacological Treatment. Curr. Med. Chem..

[3] Gallardo G., Holtzman D.M. (2019). Amyloid-β and Tau at the Crossroads of Alzheimer’s Disease. Adv. Exp. Med. Biol..

[4] Cummings J.L., Morstorf T., Zhong K. (2014). Alzheimer’s Disease Drug-Development Pipeline: Few Candidates, Frequent Failures. Alzheimer’s Res. Ther..

[5] Feldman H.H., Haas M., Gandy S., Schoepp D.D., Cross A.J., Mayeux R., Sperling R.A., Fillit H., van de Hoef D.L., Dougal S. (2014). Alzheimer’s Disease Research and Development: A Call for a New Research Roadmap. Ann. N. Y. Acad. Sci..

[6] De Strooper B. (2014). Lessons from a Failed γ-Secretase Alzheimer Trial. Cell.

[7] Swerdlow R.H., Khan S.M. (2004). A “Mitochondrial Cascade Hypothesis” for Sporadic Alzheimer’s Disease. Med. Hypotheses.

[8] Perez Ortiz J.M., Swerdlow R.H. (2019). Mitochondrial Dysfunction in Alzheimer’s Disease: Role in Pathogenesis and Novel Therapeutic Opportunities. Br. J. Pharmacol..

[9] Galla L., Redolfi N., Pozzan T., Pizzo P., Greotti E. (2020). Intracellular Calcium Dysregulation by the Alzheimer’s Disease-Linked Protein Presenilin 2. Int. J. Mol. Sci..

[10] Xie C., Aman Y., Adriaanse B.A., Cader M.Z., Plun-Favreau H., Xiao J., Fang E.F. (2020). Culprit or Bystander: Defective Mitophagy in Alzheimer’s Disease. Front. Cell Dev. Biol..

[11] McGuire P.J. (2019). Mitochondrial Dysfunction and the Aging Immune System. Biology.

[12] Sprenger H.-G., Langer T. (2019). The Good and the Bad of Mitochondrial Breakups. Trends Cell Biol..

[13] Zorov D.B., Krasnikov B.F., Kuzminova A.E., Vysokikh M.Y., Zorova L.D. (1997). Mitochondria Revisited. Alternative Functions of Mitochondria. Biosci. Rep..

[14] Meyer J.N., Leuthner T.C., Luz A.L. (2017). Mitochondrial Fusion, Fission, and Mitochondrial Toxicity. Toxicology.

[15] Hunt R.J., Bateman J.M. (2018). Mitochondrial Retrograde Signaling in the Nervous System. FEBS Lett..

[16] da Cunha F.M., Torelli N.Q., Kowaltowski A.J. (2015). Mitochondrial Retrograde Signaling: Triggers, Pathways, and Outcomes. Oxidative Med. Cell. Longev..

[17] Strobbe D., Sharma S., Campanella M. (2021). Links between Mitochondrial Retrograde Response and Mitophagy in Pathogenic Cell Signalling. Cell. Mol. Life Sci..

[18] Hauptmann S., Scherping I., Dröse S., Brandt U., Schulz K.L., Jendrach M., Leuner K., Eckert A., Müller W.E. (2009). Mitochondrial Dysfunction: An Early Event in Alzheimer Pathology Accumulates with Age in AD Transgenic Mice. Neurobiol. Aging.

[19] Stockburger C., Gold V.A.M., Pallas T., Kolesova N., Miano D., Leuner K., Müller W.E. (2014). A Cell Model for the Initial Phase of Sporadic Alzheimer’s Disease. J. Alzheimer’s Dis..

[20] Baker A.C., Ko L.-W., Blass J.P. (1988). Systemic Manifestations of Alzheimer’s Disease. AGE.

[21] Cecchi C., Fiorillo C., Sorbi S., Latorraca S., Nacmias B., Bagnoli S., Nassi P., Liguri G. (2002). Oxidative Stress and Reduced Antioxidant Defenses in Peripheral Cells from Familial Alzheimer’s Patients. Free Radic. Biol. Med..

[22] Khan T.K., Alkon D.L. (2015). Peripheral Biomarkers of Alzheimer’s Disease. J. Alzheimer’s Dis..

[23] Trushina E. (2019). Alzheimer’s Disease Mechanisms in Peripheral Cells: Promises and Challenges. Alzheimer’s Dement. Transl. Res. Clin. Interv..

[24] Gasparini L., Racchi M., Binetti G., Trabucchi M., Solerte S.B., Alkon D., Etcheberrigaray R., Gibson G., Blass J., Paoletti R. (1998). Peripheral Markers in Testing Pathophysiological Hypotheses and Diagnosing Alzheimer’s Disease. FASEB J..

[25] Pickles S., Vigié P., Youle R.J. (2018). Mitophagy and Quality Control Mechanisms in Mitochondrial Maintenance. Curr. Biol..

[26] Dodson M., Darley-Usmar V., Zhang J. (2013). Cellular Metabolic and Autophagic Pathways: Traffic Control by Redox Signaling. Free Radic. Biol. Med..

[27] Okatsu K., Saisho K., Shimanuki M., Nakada K., Shitara H., Sou Y., Kimura M., Sato S., Hattori N., Komatsu M. (2010). P62/SQSTM1 Cooperates with Parkin for Perinuclear Clustering of Depolarized Mitochondria. Genes Cells.

[28] Hernandez G., Thornton C., Stotland A., Lui D., Sin J., Ramil J., Magee N., Andres A., Quarato G., Carreira R.S. (2013). MitoTimer: A Novel Tool for Monitoring Mitochondrial Turnover. Autophagy.

[29] Ferree A.W., Trudeau K., Zik E., Benador I.Y., Twig G., Gottlieb R.A., Shirihai O.S. (2013). MitoTimer Probe Reveals the Impact of Autophagy, Fusion, and Motility on Subcellular Distribution of Young and Old Mitochondrial Protein and on Relative Mitochondrial Protein Age. Autophagy.

[30] Trudeau K.M., Gottlieb R.A., Shirihai O.S. (2014). Measurement of Mitochondrial Turnover and Life Cycle Using MitoTimer. Methods Enzymol..

[31] Walczak J., Dębska-Vielhaber G., Vielhaber S., Szymański J., Charzyńska A., Duszyński J., Szczepanowska J. (2019). Distinction of Sporadic and Familial Forms of ALS Based on Mitochondrial Characteristics. FASEB J..

[32] Drummond E., Wisniewski T. (2017). Alzheimer’s Disease: Experimental Models and Reality. Acta Neuropathol..

[33] Israel M.A., Yuan S.H., Bardy C., Reyna S.M., Mu Y., Herrera C., Hefferan M.P., Van Gorp S., Nazor K.L., Boscolo F.S. (2012). Probing Sporadic and Familial Alzheimer’s Disease Using Induced Pluripotent Stem Cells. Nature.

[34] Bialopiotrowicz E., Kuzniewska B., Kachamakova-Trojanowska N., Barcikowska M., Kuznicki J., Wojda U. (2011). Cell Cycle Regulation Distinguishes Lymphocytes from Sporadic and Familial Alzheimer’s Disease Patients. Neurobiol. Aging.

[35] Piaceri I., Nacmias B., Sorbi S. (2013). Genetics of Familial and Sporadic Alzheimer’s Disease. Front. Biosci..

[36] LaFerla F.M., Green K.N. (2012). Animal Models of Alzheimer Disease. Cold Spring Harb. Perspect. Med..

[37] Martín-Maestro P., Gargini R., García E., Perry G., Avila J., García-Escudero V. (2017). Slower Dynamics and Aged Mitochondria in Sporadic Alzheimer’s Disease. Oxidative Med. Cell. Longev..

[38] Ramamoorthy M., Sykora P., Scheibye-Knudsen M., Dunn C., Kasmer C., Zhang Y., Becker K.G., Croteau D.L., Bohr V.A. (2012). Sporadic Alzheimer’s Disease Fibroblasts Display an Oxidative Stress Phenotype. Free Radic. Biol. Med..

[39] Palotás A., Kálmán J., Laskay G., Juhász A., Janka Z., Penke B. (2002). Change of fibroblast calcium levels caused by beta-amyloid peptide in Alzheimer disease. Ideggyogy Szle.

[40] Peterson C., Ratan R.R., Shelanski M.L., Goldman J.E. (1988). Altered Response of Fibroblasts from Aged and Alzheimer Donors to Drugs That Elevate Cytosolic Free Calcium. Neurobiol. Aging.

[41] Naderi J., Lopez C., Pandey S. (2006). Chronically Increased Oxidative Stress in Fibroblasts from Alzheimer’s Disease Patients Causes Early Senescence and Renders Resistance to Apoptosis by Oxidative Stress. Mech. Ageing Dev..

[42] Wang X., Su B., Fujioka H., Zhu X. (2008). Dynamin-like Protein 1 Reduction Underlies Mitochondrial Morphology and Distribution Abnormalities in Fibroblasts from Sporadic Alzheimer’s Disease Patients. Am. J. Pathol..

[43] Birnbaum J.H., Wanner D., Gietl A.F., Saake A., Kündig T.M., Hock C., Nitsch R.M., Tackenberg C. (2018). Oxidative Stress and Altered Mitochondrial Protein Expression in the Absence of Amyloid-β and Tau Pathology in IPSC-Derived Neurons from Sporadic Alzheimer’s Disease Patients. Stem Cell Res..

[44] Zelko I.N., Mariani T.J., Folz R.J. (2002). Superoxide Dismutase Multigene Family: A Comparison of the CuZn-SOD (SOD1), Mn-SOD (SOD2), and EC-SOD (SOD3) Gene Structures, Evolution, and Expression. Free Radic. Biol. Med..

[45] Kitada M., Xu J., Ogura Y., Monno I., Koya D. (2020). Manganese Superoxide Dismutase Dysfunction and the Pathogenesis of Kidney Disease. Front. Physiol..

[46] Flynn J.M., Melov S. (2013). SOD2 in Mitochondrial Dysfunction and Neurodegeneration. Free Radic. Biol. Med..

[47] Schieber M., Chandel N.S. (2014). ROS Function in Redox Signaling and Oxidative Stress. Curr. Biol..

[48] English J., Son J.M., Cardamone M.D., Lee C., Perissi V. (2020). Decoding the Rosetta Stone of Mitonuclear Communication. Pharmacol. Res..

[49] Sebastián D., Palacín M., Zorzano A. (2017). Mitochondrial Dynamics: Coupling Mitochondrial Fitness with Healthy Aging. Trends Mol. Med..

[50] Qin W., Haroutunian V., Katsel P., Cardozo C.P., Ho L., Buxbaum J.D., Pasinetti G.M. (2009). PGC-1alpha Expression Decreases in the Alzheimer Disease Brain as a Function of Dementia. Arch. Neurol..

[51] Sheng B., Wang X., Su B., Lee H., Casadesus G., Perry G., Zhu X. (2012). Impaired Mitochondrial Biogenesis Contributes to Mitochondrial Dysfunction in Alzheimer’s Disease. J. Neurochem..

[52] Fang E.F., Hou Y., Palikaras K., Adriaanse B.A., Kerr J.S., Yang B., Lautrup S., Hasan-Olive M.M., Caponio D., Dan X. (2019). Mitophagy Inhibits Amyloid-β and Tau Pathology and Reverses Cognitive Deficits in Models of Alzheimer’s Disease. Nat. Neurosci..

[53] Li P.A., Hou X., Hao S. (2017). Mitochondrial biogenesis in neurodegeneration. J. Neurosci. Res..

[54] Tonelli C., Chio I.I.C., Tuveson D.A. (2018). Transcriptional Regulation by Nrf2. Antioxid. Redox Signal..

[55] Drabik K., Piecyk K., Wolny A., Szulc-Dąbrowska L., Dębska-Vielhaber G., Vielhaber S., Duszyński J., Malińska D., Szczepanowska J. (2021). Adaptation of Mitochondrial Network Dynamics and Velocity of Mitochondrial Movement to Chronic Stress Present in Fibroblasts Derived from Patients with Sporadic Form of Alzheimer’s Disease. FASEB J..

[56] Venditti P., Costagliola I.R., Di Meo S. (2002). H_2_O_2_ Production and Response to Stress Conditions by Mitochondrial Fractions from Rat Liver. J. Bioenerg. Biomembr..

[57] Schofield J.H., Schafer Z.T. (2021). Mitochondrial Reactive Oxygen Species and Mitophagy: A Complex and Nuanced Relationship. Antioxid. Redox Signal..

[58] Tran M., Reddy P.H. (2020). Defective Autophagy and Mitophagy in Aging and Alzheimer’s Disease. Front. Neurosci..

